# A Case Series of Aplasia Cutis Congenita and Its Management

**DOI:** 10.7759/cureus.80135

**Published:** 2025-03-06

**Authors:** Mohammed Khatija Begum, Jami Vijayashree, Aruna Bathina, Pallavi Gullipalli

**Affiliations:** 1 Dermatology, Venereology and Leprosy, Great Eastern Medical School And Hospital, Srikakulam, IND; 2 Dermatology, Venereology and Leprosy, Great Eastern Medical School and Hospital, Srikakulam, IND

**Keywords:** aplasia cutis, aplasia cutis congenita, congenital anomalies of the lower extremity, developmental defect, platelet-derived growth factor

## Abstract

Introduction: Aplasia cutis congenita is a rare condition characterized by a localized or widespread, complete or partial absence of skin at birth. In accordance with the pattern, location, underlying causes, and anomalies, Frieden divided aplasia cutis congenita into nine types. Approximately 80% of all lesions are found on the scalp. The purpose of this study is to describe uncommon instances of aplasia cutis congenita and how they are treated.

Materials and methods: Our study comprises six patients with aplasia cutis congenita belonging to either sex who attended to our dermatology outpatient department at Great Eastern Medical School and Hospital, Srikakulam during the 12-month study period from December 2023 to December 2024. This study was started after obtaining institutional ethical clearance. Patients with traumatic injuries were excluded from the study. All patients with congenital aplasia cutis were included in this study. All patients were treated with recombinant human platelet derived growth factor gel and hydrocolloid dressings for two weeks and a response was elicited.

Results: We have encountered six newborns with aplasia cutis congenita in our study, out of which four (80%) were having aplasia cutis congenita of scalp (Group I) with no other congenital anomalies, fifth case (10%) was aplasia cutis congenita with epidermolysis bullosa and dystrophic nails (Group VI - Bart syndrome) and the sixth one (10%) had aplasia cutis congenita on lower extremities without epidermolysis bullosa (Group VII).

One of the newborns was born to the mother who was taking methimazole in the first six weeks of gestation. Out of six, five babies had no consanguineous background, while one was born to parents with second-degree consanguinity.

Most common site involved was the parietal region of the scalp (80%). The smallest lesion measured 0.5x0.5 cm, while the largest was 5x3x1 cm. All the lesions showed noticeable improvement after treatment with recombinant human platelet derived growth factor 0.01% gel twice daily along with hydrocolloid dressings for two weeks.

Conclusion: Aplasia cutis congenita, being a rare disorder with less incidence is clinically diagnosed and needs to be carefully evaluated for underlying etiologies alongside other congenital anomalies co-existing with it for better management. To conclude with, our study throws a light on conservative management of aplasia cutis congenita which gave promising results minimizing complications of surgical management.

## Introduction

Aplasia cutis congenita is a rare condition characterized by a localized or widespread, complete or partial absence of skin at birth. In accordance with the pattern, location, underlying causes, and anomalies, Frieden divided Aplasia cutis congenita into nine types [1]. It is still unclear exactly how aplasia cutis congenita develops. Numerous factors, including placental infarcts, heredity, teratogenic, prenatal infections and trauma, ectodermal dysplasia, incomplete neural tube closure, and maternal intrapartum drug use, have been suspected as potential causes [2].

Approximately 80% of all lesions are found on the scalp. The most frequent location is the parietal hair whorl; in addition to affecting the skin and subcutaneous tissues, the defect may in one-third of instances spread to the underlying bone [3]. An estimate of three out of every 10,000 infants are thought to have aplasia cutis congenita [4].

Clinically, the lesions typically manifest as ulcerated, transparent, well-defined membranes that allow for the visualization of underlying structures [5]. The condition determines how it should be handled. Large, widely dispersed lesions may require surgery, but small, localized lesions may be treated conservatively [6].

The purpose of this study is to describe uncommon instances of aplasia cutis congenita and how they have been treated.

## Materials and methods

Study design

This is a prospective observational study conducted in the dermatology department of Great Eastern Medical School and Hospital, Srikakulam, Andhra Pradesh over a 12-month period from December 2023 to December 2024. This study aims to elucidate the diverse clinical presentations of Aplasia Cutis Congenita and to evaluate their treatment outcomes. The institutional ethics committee of Great Eastern Medical School and Hospital issued approval 173/IEC/GEMS&H/2023, dated December 21, 2023.

Study population

This study comprised six patients diagnosed with aplasia cutis congenita, of both sexes, who attended our dermatology outpatient department at our tertiary care hospital over a 12-month study period. The study commenced following the acquisition of institutional ethical clearance. Written informed consent was meticulously obtained from the parents. Patients presenting with traumatic injuries were excluded from the study; however, all patients with congenital aplasia cutis were inclusively enrolled.

Data collection

An elaborative history was meticulously obtained from the parents of these patients, including antenatal drug history, consanguinity, and detailed accounts of delivery events (normal vaginal delivery, cesarean section, or forceps delivery). The course of labor, whether it was eventful or uneventful, was thoroughly documented, along with any pertinent history of similar complaints in siblings and any significant prenatal infections. The birth weight was meticulously recorded.

Comprehensive general, systemic, and cutaneous examinations were diligently conducted, with a keen focus on identifying other sites of involvement and evaluating for additional congenital anomalies. High-quality clinical photographs were taken, and treatment was administered using 0.01% recombinant human platelet-derived growth factor gel. This advanced treatment was accompanied by sterile dressings and hydrocolloid dressings, and stringent wound care was maintained under close supervision for six weeks to monitor the clinical response.

Statistical analysis

Data were analyzed using comprehensive descriptive statistics to provide a detailed and thorough summary of patient demographics, clinical features, and treatment outcomes.

## Results

Case 1

Our first case involves a 10-day-old female infant, weighing 2 kg, who was born full-term via spontaneous normal vaginal delivery. The infant was brought in by her parents from Amadalavalasa, Srikakulam district, with a complaint of an ulcer on the occipital region of her scalp since birth. The pregnancy, labor, and delivery were uneventful, and the mother had not taken any significant medications during pregnancy. There were no similar complaints among the infant’s siblings or family members.

**Figure 1:**
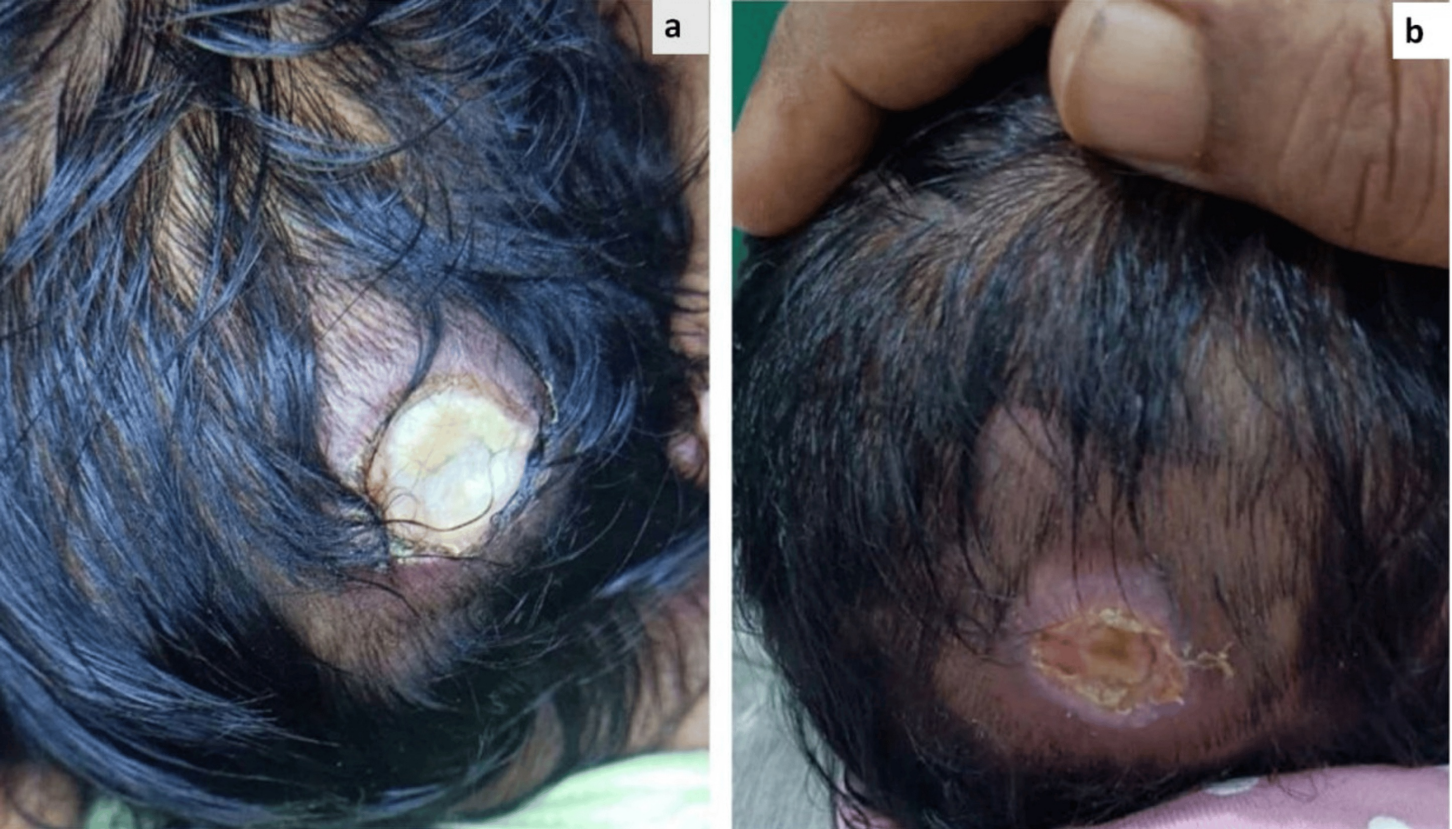
(a) Aplasia cutis congenita of scalp (Group 1); defect over occipital region measuring 1x1 cm covered with slough. (b) Defect healed with red granulation tissue after two weeks of application with recombinant human platelet derived growth factor gel.

Upon examination, the infant's vital signs and systemic assessment were normal. The cutaneous examination revealed a single, well-defined round ulcer (Figure 1a) measuring 1x1 cm on the occipital region, with a raised erythematous border covered in yellow slough. No other congenital anomalies were noted. An ultrasound examination of the cranium appeared normal.

The treatment regimen included daily dressings with Betadine, followed by the removal of slough and the application of recombinant human platelet-derived growth factor gel 0.01% to the ulcer twice daily. This was then covered with hydrocolloid dressings. The ulcer showed signs of epithelialization within a couple of weeks. The patient was followed up every fortnight for six weeks, after which the ulcer had completely healed, leaving an atrophic hairless scar (Figure 1b). A favorable prognosis was provided to the parents, as the lesion was small and there were no underlying defects, resulting in their satisfaction with the outcome.

Case 2

Our second case involves a five-day-old male infant, weighing 2.4 kg, who was born at full-term via spontaneous normal vaginal delivery. The infant was brought in by his parents from Parlakhemundi, Gajapati district, Odisha, with a complaint of a skin defect on the scalp since birth. The antenatal, natal, and postnatal periods were uneventful, with no significant family or medication history.

**Figure 2:**
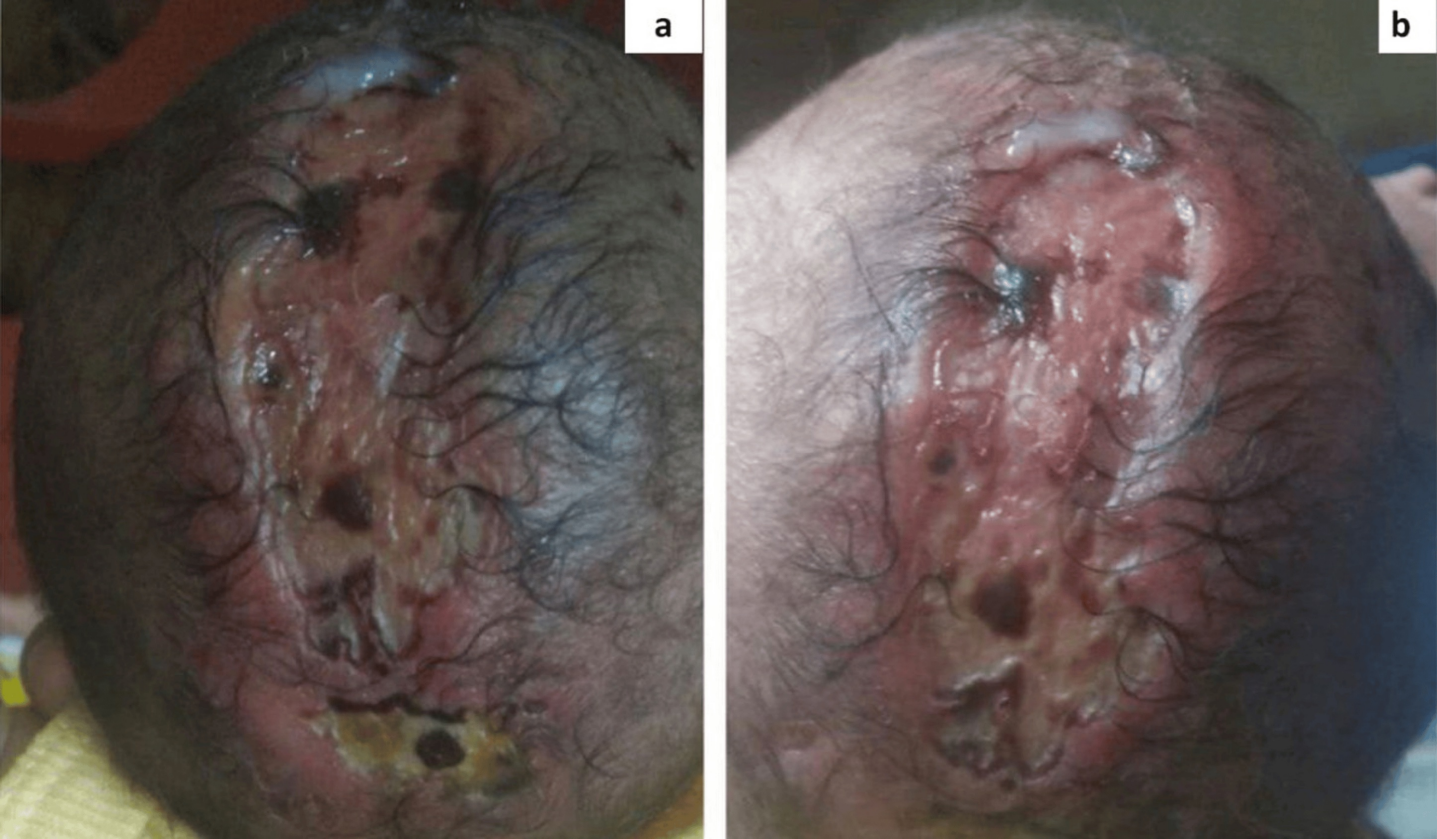
(a) Aplasia cutis congenita of scalp (Group I); larger defect measuring 5x3x1 cm extending from vertex to occipital region covered with necrotic slough. (b) Defect over scalp started to heal after seven days of application with recombinant human platelet derived growth factor gel.

Upon examination, a single, well-defined, irregularly shaped ulcer measuring 5x3x1 cm was observed, extending from the vertex to the occipital region of the scalp, with serosanguinous discharge (Figure 2a). No other congenital anomalies were noted. An ultrasound examination of the cranium indicated no underlying defects.

For treatment, daily dressings with Betadine were recommended. Necrotic slough was removed daily, followed by the application of recombinant human platelet-derived growth factor gel 0.01% to the defect twice a day, which was then covered with hydrocolloid dressings. The defect began to heal within 14 days (Figure 2b). The parents were advised to bring the child in for follow-up every fortnight, and treatment continued for six weeks, showing significant progress and culminating in complete re-epithelialization of the defect. The parents expressed satisfaction with the results.

Case 3

The third case involves a four-day-old male infant weighing 2 kg, born at 39 weeks of gestation through a spontaneous normal vaginal delivery. The parents, who are from Palakonda in the Parvathipuram district, brought the child to the dermatology department of our hospital due to the absence of skin on the anterior abdomen and the right side of the face since birth. The pregnancy, labor, and delivery were uneventful, and the antenatal medication history was not significant. The infant cried immediately after birth.

**Figure 3:**
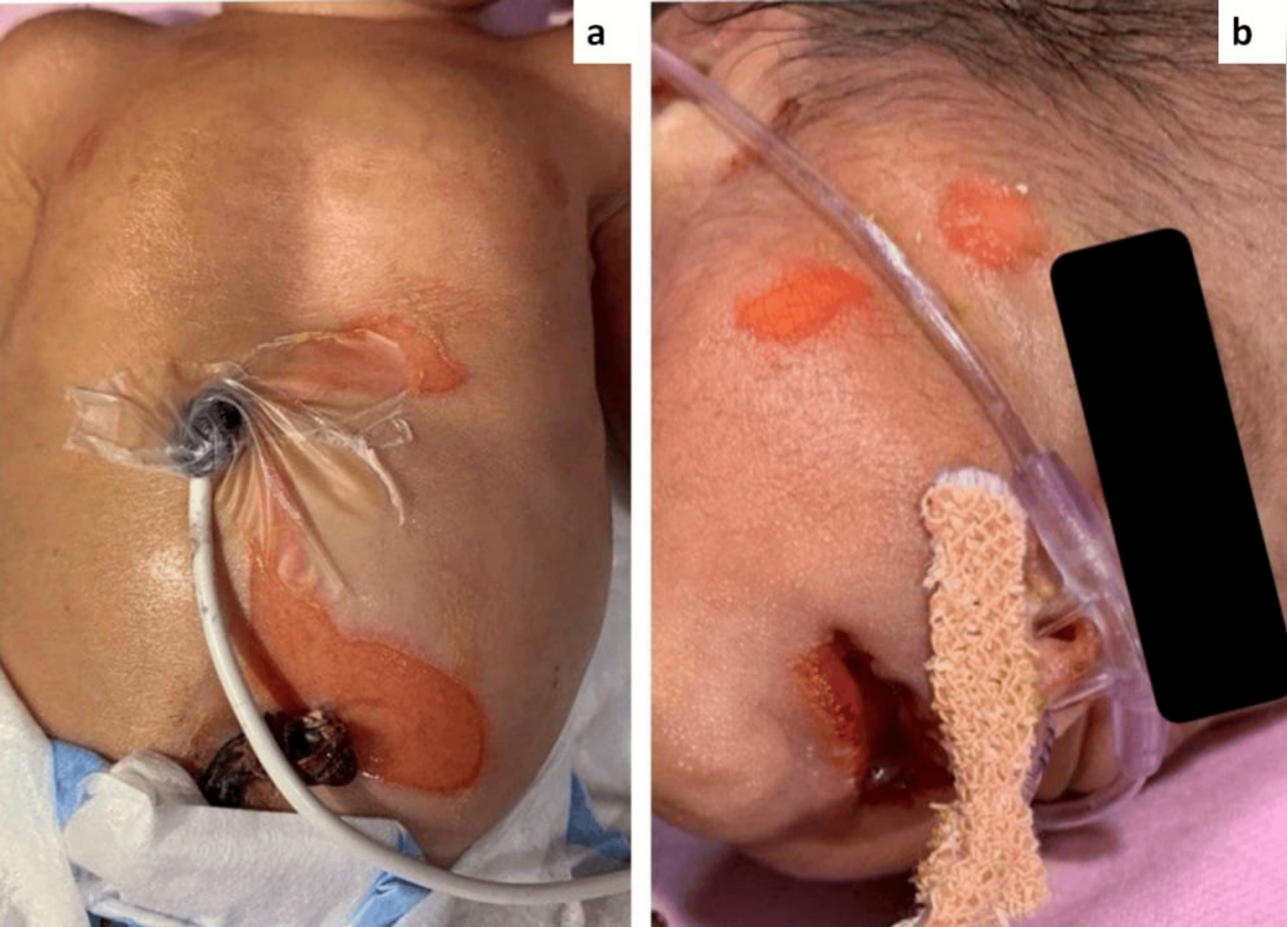
(a) Aplasia cutis congenita (Group VII); absence of skin over anterior abdomen extending from xiphisternum to periumbilical area. (b) Absence of skin over right side of face with underlying vascular structures being easily visualized.

Upon examination, there was a complete absence of skin on the right side of the face and the anterior abdomen (Figure 3a). The abdomen showed peeling skin and was covered with a red, glistening membrane, extending from the xiphoid process to the periumbilical area (Figure 3b). No other congenital abnormalities were noted.

The parents were advised to avoid friction and the use of adhesives, and to maintain a sterile environment with appropriate ambient room temperature. We recommended the application of recombinant human platelet-derived growth factor gel (0.01%) over the lesions twice a day, followed by coverage with hydrocolloid dressings for 14 days. After this period, re-epithelialization began. The parents were instructed to return with the child for follow-up every two weeks until six weeks of treatment. After this time, the defect had completely healed, and the parents expressed their satisfaction with the outcome.

The prognosis for the child was good, as no underlying abnormalities were detected and the lesions began healing within 14 days. The parents were carefully educated about the fragile nature of the infant's skin, the need for gentle handling, and the self-healing properties of the lesions.

Case 4

A female infant, born prematurely at 35 weeks gestation and weighing 1.4 kg, was delivered via lower segment cesarean section to a mother from Narasannapeta, Srikakulam district. The mother reported no significant antenatal, natal, or postnatal complications, and the infant's siblings exhibited no similar conditions. Upon birth, the infant presented with a complete absence of skin on both lower legs and experienced blistering on otherwise normal skin due to minor trauma or friction from adhesive bandages. At birth, the infant cried promptly, displayed good muscle tone, and moved all four limbs.

**Figure 4:**
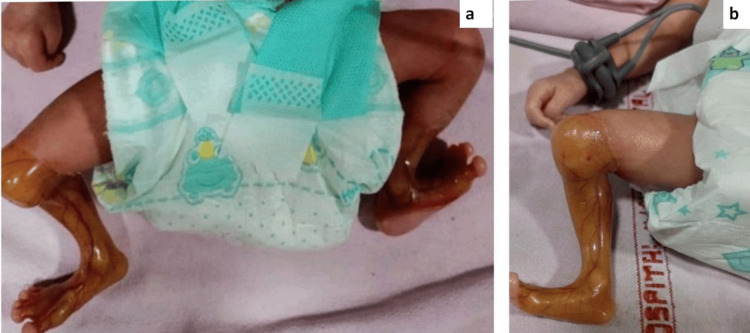
(a) Aplasia cutis congenita (Group VI - Bart syndrome); absence of skin over bilateral lower legs extending from knees to dorsal and medial plantar aspect of feet. (b) Lower leg lesion covered by a red ultrathin translucent glistening membrane with underlying vascular structures being easily visualized.

Clinical examination revealed an absence of skin extending from the knees to the feet bilaterally (Figure 4a), characterized by sharply defined borders and a translucent membrane exposing the underlying vascular structures (Figure 4b). Additional findings included toenail dystrophy and premature genitalia. A 2D echocardiogram identified transposition of the great arteries (TGA), a congenital heart defect. An abdominal ultrasound yielded normal results.

The infant was treated with recombinant human platelet-derived growth factor gel (0.01%) applied twice daily, followed by hydrocolloid dressings. Significant improvement was observed after two weeks, with re-epithelialization achieved by six weeks. We informed the parents of a positive prognosis due to the mild extent and severity of the lesions.

Case 5

**Figure 5:**
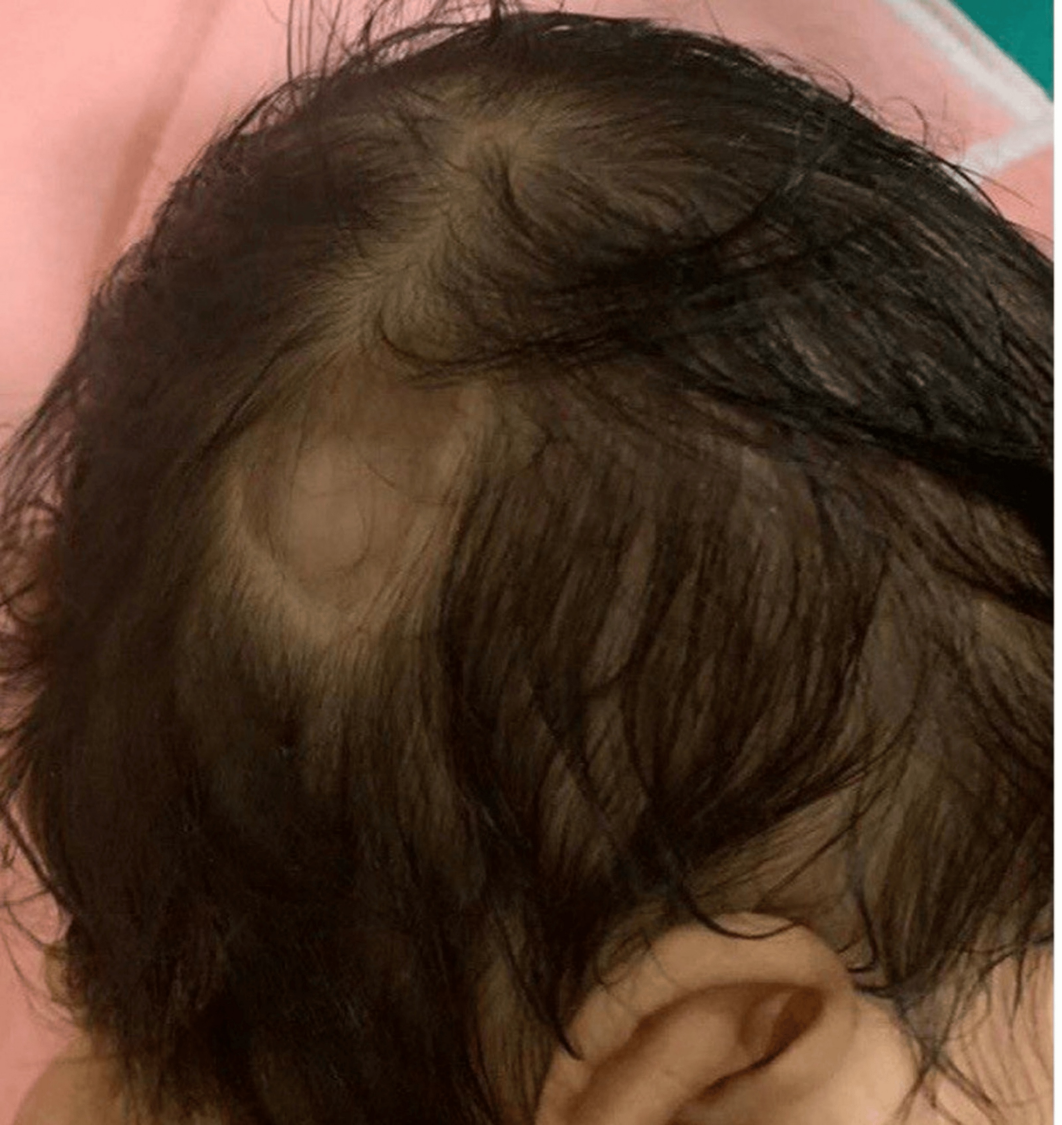
Aplasia cutis congenita of scalp (Group I); defect over parieto-occipital region of scalp measuring 2X2 cm.

A three-day-old male infant, weighing 2.4 kg and born full-term via spontaneous normal vaginal delivery to consanguineous parents (second degree) from Gunupur, Rayagada district, Orissa, presented to the dermatology department with an absence of skin over the right parieto-occipital region of the scalp measuring 2x2 cm, had been present since birth (Figure 5).

The infant cried immediately after birth, exhibited good muscle tone, and had an uneventful antenatal, natal, and postnatal history. There was no history of teratogenic drug exposure from the mother, and no other congenital anomalies were observed. General examination, vital signs, and systemic examination were all normal.

The patient was treated with recombinant human platelet-derived growth factor gel 0.01%, applied twice daily to the scalp defect, followed by hydrocolloid dressings for two weeks. Re-epithelialization of the defect began after a couple of weeks. The child was brought in for follow-up visits every two weeks for a total of six weeks, during which the treatment continued. The defect eventually healed completely. The parents were informed about the favorable prognosis, as the lesion was small and without any underlying defects. They expressed satisfaction with the results.

Case 6

A five-day-old female infant, weighing 2.3 kg and born full-term via spontaneous normal vaginal delivery, was brought to our dermatology outpatient department by her parents from Nidadavolu, East Godavari district. The parents reported a congenital absence of skin over the right parietal region of the scalp, measuring 0.5x0.5 cm, present since birth.

The infant cried immediately after birth, exhibited good muscle tone, and had an uneventful antenatal, natal, and postnatal history. The mother had a history of hyperthyroidism and was on antithyroid medication, methimazole, during the first six weeks of her pregnancy. No other congenital anomalies were observed, and general examination, vital signs, and systemic examination were all within normal limits.

Treatment involved the application of recombinant human platelet-derived growth factor gel (0.01%) to the affected area twice daily. Notable epithelialization was observed within two weeks. Follow-up visits were scheduled every two weeks until the treatment period reached six weeks. The scalp defect gradually healed with an atrophic scar. The parents were informed about the favorable prognosis of aplasia cutis of the scalp, as it generally responds well to treatment, indicating a positive outcome.

The comprehensive clinical profiles of six patients diagnosed with aplasia cutis congenita, including distribution of lesions, associations with other congenital anomalies, consanguinity, teratogenic history, and treatment outcomes, are summarized in Table 1.

**Table 1:** Clinical profile and treatment outcomes of aplasia cutis congenita patients.

Patient	Age	Sex	Sites involved	Consanguineous history	Teratogen history	Associated anomalies	Response to treatment
Case 1	10 days	Female	Occipital region	No	No	No	Good
Case 2	5 days	Male	Vertex, occipital region	No	No	No	Moderate
Case 3	4 days	Male	Anterior abdomen, right side of face	No	No	No	Moderate
Case 4	1 day	Female	Bilateral lower legs and feet	No	No	Epidermolysis bullosa, toenail dystrophy present, premature genitalia, transposition of great arteries	Moderate
Case 5	3 days	Male	Right parieto-occipital region	Yes (second degree)	No	No	Good
Case 6	5 days	Female	Right parietal region of scalp	No	Intake of methimazole present	No	Good

## Discussion

Aplasia cutis congenita is a rare birth defect. Since its first description, over 500 cases have been reported; nevertheless, the exact frequency of this typically benign condition is unknown due to considerable underreporting. For instance, there are estimates of incidence that range from 0.3% to roughly three occurrences per 10,000 births [7]. Although deficiencies may also develop on the face, trunk, or limbs, occasionally symmetrically, the majority of aplasia cutis congenita lesions occur on the vertex of scalp immediately lateral to the midline. Only the epidermis and upper dermis may be affected, leaving little alopecic scarring; alternatively, the defect may spread into the deep dermis, subcutaneous tissue, and in rare cases, the periosteum, skull, and dura.

Frieden developed a nine-group classification system for aplasia cutis congenita that is based on the number and location of lesions as well as the existence or lack of related deformities [1].

Group I

This group comprises aplasia cutis congenita of the scalp without any other abnormalities [8]. The scalp is the site of around 86% of all solitary lesions. Especially in cases of membranous aplasia cutis, a collar of hair is frequently observed surrounding the lesion. It may be sporadic [9] or autosomal dominant [10].

Group II

This group comprises limb abnormalities associated with scalp aplasia cutis congenita. Adams-Oliver syndrome [11-12] is a unique condition where solitary midline scalp anomalies are linked to abnormalities in distal limb reduction. The inheritance patterns of Adams-Oliver syndrome are both autosomal dominant and autosomal recessive. Hypoplastic or absent distal phalanges are the most prevalent limb deformity. Cutis marmorata telangiectatica congenita, hemangiomas, cranial arteriovenous malformation, congenital heart problems, skin tags, supernumerary nipples, and woolly hair are some additional abnormalities that may be present.

Group III

This group comprises aplasia cutis congenita of the scalp with sebaceous (organoid) and epidermal nevi [13] which typically affects the scalp next to the aplasia cutis. Seizures, intellectual impairment, corneal opacities, and eyelid colobomas are among the neurologic and ocular symptoms that are typical of epidermal nevus syndrome that some individuals have experienced. Aplasia cutis congenita, pigmented nevus, limbal dermoid, nevus sebaceous, and central nervous system abnormalities have all been grouped together as SCALP (nevus sebaceus, central nervous system malformations, aplasia cutis congenita, limbal dermoid, pigmented nevus) syndrome [13]. The inheritance is sporadic [14].

Group IV

This group comprises congenital aplasia cutis that is covering more profound embryological abnormalities [15]. Leptomeningeal angiomatosis, cranial stenosis, spinal dysraphism, gastroschisis, omphalocele, meningomyelocele, and porencephaly are a few examples. Frequently, the scalp lesions that cover neural tube abnormalities have a hair collar.

Group V

This group comprises aplasia cutis congenita linked to placental infarct or fetal papyraceous [16]. The death of a twin fetus in the late first or early second trimester results in a fetal papyraceous, which is discovered at the time of delivery. There is widespread limb and truncal aplasia cutis congenita in the surviving fetus.

Group VI

This group comprises the condition, commonly known as Bart syndrome, which is aplasia cutis congenita linked to epidermolysis bullosa (EB) [17]. Any form of EB, whether it be dystrophic, junctional, or simple, can present with aplasia cutis congenita. Aplasia cutis congenita, which typically affects the lower extremities, is described in numerous cases.

Group VII

This group comprises aplasia cutis congenita localized to the extremities without epidermolysis bullosa [18-19].

Group VIII

This group comprises a teratogen-induced aplasia cutis congenita. A few instances of aplasia cutis congenita have been connected to methimazole exposure, in the treatment of maternal thyrotoxicosis during pregnancy, or intrauterine infection with the herpes simplex or varicella zoster viruses [20-21].

Group IX

This group comprises a congenital aplasia cutis linked to malformation syndromes [22]. Numerous syndromes, such as trisomy 13 (Patau syndrome) with large membranous scalp defects, 4p-(Wolf-Hirschhorn) syndrome with midline scalp defects, Setleis syndrome with bitemporal aplasia cutis congenita and abnormal eyelashes, Johanson-Blizzard syndrome with stellate scalp defects, and focal dermal hypoplasia (Goltz syndrome), have been reported to exhibit aplasia cutis congenita [23].

The prognosis for aplasia cutis congenita is usually extremely excellent. If the defect is small, recovery is uneventful; the epithelium will progressively epithelialize and a hairless, atrophic scar will emerge over a few weeks. Full-thickness anomalies of the dura, scalp, and skull are associated with a mortality risk of above 50%. Individuals who have the rarer, larger scalp anomalies are at risk of dying from infection and hemorrhage. Widespread aplasia cutis congenita of the scalp may be associated with an increased risk of sagittal sinus thrombosis. These factors may make surgical correction necessary for significant, full-thickness scalp abnormalities.

Blionas et al. reported two cases of aplasia cutis congenita of the scalp [24]. In the first case, a child with a smaller defect was managed conservatively with Betadine dressings and bacitracin ointment. Similarly, our cases showed significant improvement with conservative management involving platelet-derived growth factor. The second child, who had a larger defect, required surgical intervention, and a single scalp flap was placed over the affected area.

Magliah & Alghamdi reported a case of a 45-day-old boy diagnosed with aplasia cutis congenita of the scalp [25]. He was born full-term via spontaneous vaginal delivery to parents who were second-degree cousins. The condition was successfully treated with topical mupirocin ointment. Similarly, in our fifth case, another baby born to second-degree consanguineous parents was successfully treated conservatively with platelet-derived growth factor.

The impact of consanguinity on the incidence of congenital malformations was described by Tayebi et al., who reported that the rate of congenital malformations was 2% among neonates from non-consanguineous marriages compared to 7% from consanguineous marriages [26]. This supports our fifth case of aplasia cutis, where the newborn was born to second-degree consanguineous parents. Moreover, a history of congenital malformations was more common in siblings from consanguineous marriages compared to those from non-consanguineous marriages.

Karg et al. documented a case in which a patient was exposed to the anti-thyroid medication methimazole during the initial six weeks of gestation [21]. The patient was born prematurely and presented with scalp and skull defects, as well as facial asymmetry. Similarly, in our sixth case, a mother who received methimazole during the first six weeks of pregnancy gave birth to a newborn diagnosed with aplasia cutis congenita.

## Conclusions

Aplasia cutis congenita is a rare disorder that should be diagnosed through clinical evaluation. It is important to carefully assess for underlying causes and other congenital anomalies. Fortunately, the prognosis for aplasia cutis congenita is generally excellent. When the defect is small, recovery proceeds smoothly, involving gradual epithelialization and the formation of an atrophic scar. We believe that the most effective way to manage aplasia cutis congenita is through a conservative approach. In all our cases, we utilized recombinant human platelet-derived growth factor 0.01% gel, which facilitated rapid healing and epithelialization, complemented by careful sterile dressings and wound care.

Surgical intervention may be considered based on clinical factors such as the size of the lesion, depth of involvement, prematurity, and potential scarring concerns. In conclusion, we firmly believe that a conservative approach maximizes the newborn’s natural ability for rapid regeneration while minimizing complications and avoiding unintended surgical consequences.
